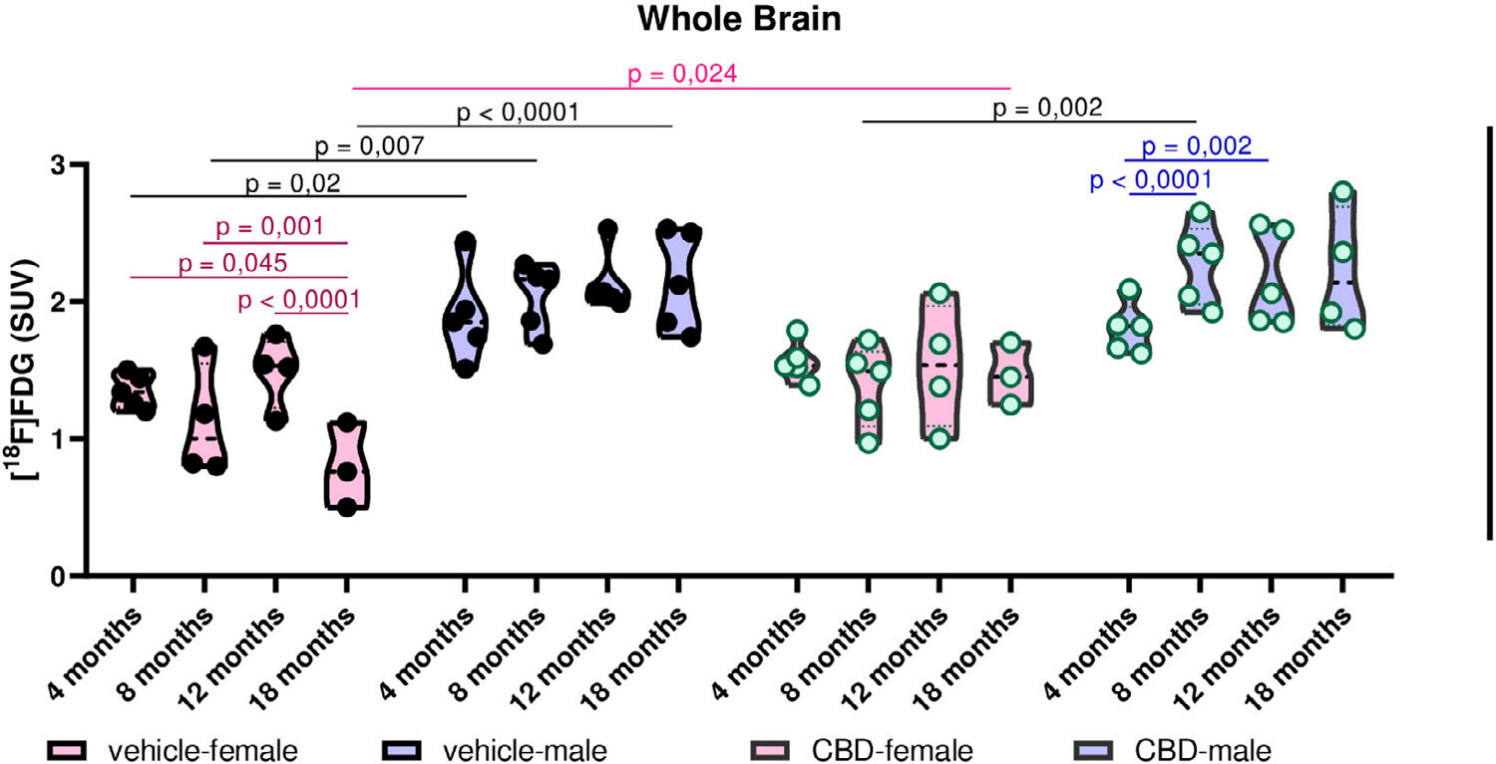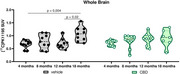# Evaluation of the effect of cannabidiol treatment on [^18^F]FDG and [^11^C]PK11195 uptake in an animal model for Alzheimer's disease

**DOI:** 10.1002/alz70856_100426

**Published:** 2025-12-24

**Authors:** Jean Marques Brizola, Chiara Maria Righini, Carlos Alberto Buchpiguel, Lidia Emmanuela Wiazowski Spelta, Daniele de Paula Faria

**Affiliations:** ^1^ University of São Paulo Medical School, São Paulo, São Paulo, Brazil; ^2^ Universidade de São Paulo, São Paulo, SP, Brazil

## Abstract

**Background:**

Cannabidiol (CBD) has well‐described anti‐inflammatory and neuroprotective properties. Emerging preclinical research has explored the potential of this cannabinoid as a therapeutic agent to delay or prevent the onset of symptoms and pathophysiological features of neurodegenerative processes, such as Alzheimer's disease (AD). Therefore, the aim of this study was to evaluate the effect of CBD treatment during aging of an animal model for AD using positron emission tomography (PET).

**Method:**

Male and female 3xTg‐AD mice (ethics committee 1811/2022) were divided into control and CBD groups. At 7 months‐old, the animals were treated with CBD (20 mg/kg) or vehicle for 30 days. At 4, 8, 12 and 18 months‐old, [^18^F]FDG and [^11^C]PK11195 PET images were acquired to assess brain metabolism and neuroinflammation. The standardized uptake value (SUV) was calculated for the whole brain. The behavioral tests of novel object recognition (NOR) and elevated plus maze (EPM) were performed to assess memory, exploratory behavior and anxiety.

**Result:**

There was a sex effect on [^18^F]FDG uptake, with vehicle‐males showing higher uptake than vehicle‐females at 4 (*p* = 0.02), 8 (*p* = 0.007), and 18 months (*p* <0.0001). Vehicle‐females had reduced [^18^F]FDG uptake at 18 months compared to all other ages (18 months vs: 4: *p* = 0.045; 8: *p* = 0.001; 12: *p* <0.0001). In the CBD group, [^18^F]FDG uptake was higher in males than in females at 8 months (*p* = 0.002). CBD‐males also had increased uptake from 4 to 8 months (*p* <0.0001) and to 12 months (*p* = 0.002). In addition, CBD‐females had higher [^18^F]FDG uptake than vehicle‐females at 18 months (*p* = 0.024). No sex effect was observed for [^11^C]PK11195. Increased uptake was observed in the control group at 18 months compared to 4 months (*p* = 0.004) and 12 months (*p* = 0.02). No significant differences were observed in the behavioral assessments.

**Conclusion:**

Our study demonstrated prominent sex differences in brain metabolism and provided evidence of a possible neuroprotective role of CBD in mitigating neuroinflammatory events associated with aging. Furthermore, PET proved to be a more sensitive tool for detecting aging‐induced changes in the 3xTg‐AD model compared to behavioral assessments.